# Supplementary material for: Developmental divergence of sensory stimulus representation in cortical interneurons
Source: Nat Commun. 2020 Nov 12;11:5729. doi: 10.1038/s41467-020-19427-z (PMC7661508; doi:10.1038/s41467-020-19427-z)
Supplement: Supplementary file 10 — Reporting Summary [file 41467_2020_19427_MOESM10_ESM.pdf]

nature research

Corresponding author(s): Theofanis Karayannis

Last updated by author(s): Oct 5, 2020

Reporting Summary

Nature Research wishes to improve the reproducibility of the work that we publish. This form provides structure for consistency and transparency in reporting. For further information on Nature Research policies, see Authors & Referees and the Editorial Policy Checklist.

Statistics

For all statistical analyses, confirm that the following items are present in the figure legend, table legend, main text, or Methods section.

n/a	Confirmed	
☐	☒	The exact sample size (*n*) for each experimental group/condition, given as a discrete number and unit of measurement
☐	☒	A statement on whether measurements were taken from distinct samples or whether the same sample was measured repeatedly
☐	☒	The statistical test(s) used AND whether they are one- or two-sided<br>*Only common tests should be described solely by name; describe more complex techniques in the Methods section.*
☐	☒	A description of all covariates tested
☐	☒	A description of any assumptions or corrections, such as tests of normality and adjustment for multiple comparisons
☐	☒	A full description of the statistical parameters including central tendency (e.g. means) or other basic estimates (e.g. regression coefficient) AND variation (e.g. standard deviation) or associated estimates of uncertainty (e.g. confidence intervals)
☐	☒	For null hypothesis testing, the test statistic (e.g. *F*, *t*, *r*) with confidence intervals, effect sizes, degrees of freedom and *P* value noted<br>*Give P values as exact values whenever suitable.*
☒	☐	For Bayesian analysis, information on the choice of priors and Markov chain Monte Carlo settings
☒	☐	For hierarchical and complex designs, identification of the appropriate level for tests and full reporting of outcomes
☐	☒	Estimates of effect sizes (e.g. Cohen's *d*, Pearson's *r*), indicating how they were calculated

Our web collection on statistics for biologists contains articles on many of the points above.

Software and code

Policy information about availability of computer code

Data collection	2P: Scanimage (2016)<br>Ephys: Clampex (10.7.03)<br>mesoSPIM: custom software (Version: 0.1.2; https://github.com/mesoSPIM/mesoSPIM-control/releases/tag/0.1.2)<br>Confocal: Fluoview<br>Slide Scanner: ZEN 2 Slidescan
Data analysis	Fiji/Image J(1.52i) was used for standard processing of images (adjust histograms, downsize, crop, reslice, rotate)<br>Imaris (9.2.0) was used for creating videos and segmenting thalamic nuclei<br>A custom Matlab (R2016b) code was used to analyse the in-vivo 2P data<br>A custom deep-neural network (DeNeRD), a custom Matlab (R2016b) code & Boxy SVG was used to analyse the rabies virus data<br>Calmpfit (10.7.03) and Matlab (R2016b) was used to analyse the electrophysiology data<br>A custom Matlab (R2016b) code was used to analyse the co-localization of tdtomato and VGlut2<br>All statistics were calculated in Matlab (R2016b)

For manuscripts utilizing custom algorithms or software that are central to the research but not yet described in published literature, software must be made available to editors/reviewers. We strongly encourage code deposition in a community repository (e.g. GitHub). See the Nature Research guidelines for submitting code & software for further information.

Data

Policy information about availability of data

All manuscripts must include a data availability statement. This statement should provide the following information, where applicable:

- Accession codes, unique identifiers, or web links for publicly available datasets
- A list of figures that have associated raw data
- A description of any restrictions on data availability

Data is available upon request. Source data is provided with this paper.

Field-specific reporting

Please select the one below that is the best fit for your research. If you are not sure, read the appropriate sections before making your selection.

☒ Life sciences ☐ Behavioural & social sciences ☐ Ecological, evolutionary & environmental sciences

For a reference copy of the document with all sections, see nature.com/documents/nr-reporting-summary-flat.pdf

Life sciences study design

All studies must disclose on these points even when the disclosure is negative.

Sample size	No statistical methods were used to determine sample size.<br>We used 3 animals per cell type and age group for in-vivo 2P Ca2+ imaging. This is comparable with sample sizes commonly used in the field (see recent papers using 2P imaging of developing cortical GABAergic neurons: Modol et al., Neuron,2020 and Duan et al., Neuron, 2020)<br>A minimum of 3 animals were used per cell type and age group for Glutamate uncaging experiments. This is comparable with sample sizes within the field (see for example Anastiasiades et al. Nat. Comm., 2015)<br>A minimum of 3 animals per cell type and age group for anatomical experiments experiments. This is comparable with sample sizes used in the field (see for example Che et al., Neuron, 2018)
Data exclusions	in-vivo 2P imaging: one animal was excluded from analysis because the cells were hyper-excitable<br>rabies-tracing: brains were excluded from anaylsis if the injection site was not in the barrel field<br>electrophysiology and photostimulation: recordings were excluded if access resistance changed with more than 20% or rose above 25MOhm<br>photostimulation: recordings were excluded if 3 repetitions of stimulation were not possible for each grid pattern spot.<br>Exclusion criteria were pre-established and are standard in the field.
Replication	In-vivo, electrophysiology and anatomical data were collected by three different researchers. All data was replicated across a minimum of 3 animals. All attempts at replication were successful.
Randomization	Animals were bred so that every pup from a specific mother carried the same genetic modification (exp. VIPCre-Ai14 or VIPCre-HTB). Animals were then randomly selected from within their litter.
Blinding	Blinding was not possible since all the animals from a specific litter carried the same genetic modification. In-vivo 2photon and in-vitro electrophysiology experiments additionally required targeting of tdtomato expressing cells.

Reporting for specific materials, systems and methods

We require information from authors about some types of materials, experimental systems and methods used in many studies. Here, indicate whether each material, system or method listed is relevant to your study. If you are not sure if a list item applies to your research, read the appropriate section before selecting a response.

Materials & experimental systems

n/a	Involved in the study	
☐	☒	Antibodies
☒	☐	Eukaryotic cell lines
☒	☐	Palaeontology
☐	☒	Animals and other organisms
☒	☐	Human research participants
☒	☐	Clinical data

Methods

n/a	Involved in the study	
☒	☐	ChIP-seq
☒	☐	Flow cytometry
☒	☐	MRI-based neuroimaging

Antibodies

Antibodies used	Rabbit anti VGlut2 (135 402, Synaptic Systems)<br>Chicken anti GFP (ab13970, abcam)

	Donkey anti Rabbit Alexa Fluor 488 (A21206, Invitrogen)<br>Donkey anti Chicken Alexa Fluor 488 (703-545-155, Jackson ImmunoResearch)
Validation	The VGlut2 antibody has been used to label thalamic terminals in the mouse brain in several publications, for example: Rousseaux MWC et al., Neuron. 2018; Jaarsma D et al., J Compt Neurol., 2018; Baquero AF et al., J Neurosci., 2015 (exhaustive list available on www.sysy.com)<br>The GFP antibody has been used to enhance endogenous GFP signal in several publication, including: Yu et al., Nat. Commun, 2020, Wilson et al., Nat. Commun., 2020, Murphy-Royal et al., Nat. Commun., 2020 (exhaustive list available on www.abcam.com)

Animals and other organisms

Policy information about studies involving animals; ARRIVE guidelines recommended for reporting animal research

Laboratory animals	Animal lines used in this study are: VIP-IRES-Cre (Viptm1(cre)Zjh/J), SST-IRES-Cre (Ssttm2.1(cre)Zjh/J), Ai14 (B6;129S6-Gt(ROSA)26Sortm14(CAG-tdTomato)Hze/J)and HTB (Gt(ROSA)26Sortm1(CAG-neo,-HTB)Fhg). All lines were maintained on a C57BL/J6 background.<br>For in-vivo 2P imaging, electrophysiology and immunohistochemistry either VIP-IRES-Cre or SST-IRES-Cre was combined with with Ai14. Animals used in these experiments were either between P8 and P12 or older than P21. Both males and females were used.<br>For the rabies virus tracings either VIP-IRES-Cre or SST-IRES-Cre was combined with HTB. Animals were injected either at P4-5 or P15 and sacrificed 7 days later (P11-12 and P22). Both males and females were used.
Wild animals	This study does not involve wild animals
Field-collected samples	This study does not contain field-collected samples
Ethics oversight	All animal experiments were performed according to the ethical guidelines of the veterinary office of the canton of Zurich and the University of Zurich and were approved by the veterinary office of the canton of Zurich.

Note that full information on the approval of the study protocol must also be provided in the manuscript.
